# Heterogeneous Damage to the Olfactory Epithelium in Patients with Post-Viral Olfactory Dysfunction

**DOI:** 10.3390/jcm12155007

**Published:** 2023-07-29

**Authors:** Shu Kikuta, Bing Han, Tatsuya Yamasoba

**Affiliations:** 1 Department of Otolaryngology-Head and Neck Surgery, Faculty of Medicine, Nihon University, 30-1, Oyaguchi Kami-cho, Itabashi-ku, Tokyo 173-8610, Japan; 2 Department of Otolaryngology-Head and Neck Surgery, Faculty of Medicine, University of Tokyo, 7-3-1 Hongo, Bunkyo-ku, Tokyo 113-8655, Japan; iceicekoi@163.com (B.H.); tyamasoba-tky@umin.ac.jp (T.Y.)

**Keywords:** olfactory sensory neuron, olfactory dysfunction, post-viral olfactory dysfunction, olfactory functional test

## Abstract

Objectives: Post-viral olfactory dysfunction (PVOD) is a neurogenic disorder caused by a common cold virus. Based on the homology of deduced amino acid sequences, olfactory sensory neurons (OSNs) in both mice and humans express either class I or class II odorant receptor genes encoding class I and class II OSNs. The purpose of this study was to determine whether OSN damage in PVOD occurs uniformly in both neuron types. Materials and methods: The characteristics of PVOD patients were compared with those of patients with chronic rhinosinusitis (CRS) or post-traumatic olfactory dysfunction (PTOD). Briefly, subjects underwent orthonasal olfaction tests using five different odors (T&T odors) and a retronasal olfaction test using a single odor (IVO odor). The regions in the mouse olfactory bulb (OB) activated by the T&T and the IVO odors were also examined. Results: Multivariate analysis of 307 cases of olfactory dysfunction (PVOD, 118 cases; CRS, 161 cases; and PTOD, 28 cases) revealed that a combination of responses to the IVO odor, but not to the T&T odors, is characteristic of PVOD, with high specificity (*p* < 0.001). Imaging analysis of GCaMP3 mice showed that the IVO odor selectively activated the OB region in which the axons of class I OSNs converged, whereas the T&T odors broadly activated the OB region in which axons of class I and class II OSNs converged. Conclusions: A response to T&T odors, but not IVO odor, in PVOD suggests that class I OSNs are injured preferentially, and that OSN damage in PVOD may occur heterogeneously in a neuron-type-dependent manner.

## 1. Introduction

Post-viral olfactory dysfunction (PVOD) is characterized by hyposmia and anosmia, which occur after upper respiratory tract infection by a common cold virus. PVOD, the major category of olfactory disorders, is present in approximately 10–20% of patients with olfactory impairment [1,2,3,4]. The degree of PVOD is generally severe, with substantial functional anosmia [5]. Furthermore, this severe olfactory impairment persists for many years after the resolution of rhinorrhea and congestion [6]. Despite the fact that olfactory loss reduces quality of life, the pathophysiology of PVOD is not well-understood.

Olfactory functional tests are divided into two categories based on whether the odor stimulates the orthonasal or the retronasal pathway. In Japan, two olfactory tests are performed frequently: the T&T (Takagi and Toyoda) olfactometry test, which has an orthonasal stimulus, and the intravenous olfactory (IVO) test, which has a retronasal stimulus [7,8]. In the T&T test, five odorants (β-phenylethyl alcohol, methyl cyclopentenolone, isovaleric acid, γ-undecalactone, skatol; defined as T&T odors) are used as the stimulus. In the IVO test, prosultiamine (defined as the IVO odor) is administered intravenously to activate olfactory sensory neurons (OSNs) of the alveoli via the retronasal pathway [8]. These tests have been widely used to estimate the degree of olfactory impairment; however, the efficacy of the two tests has not been established in other contexts.

The olfactory epithelium (OE) is divided into zones I, II, III, and IV (from the dorsomedial to ventral region), in which OSNs are densely packed. In many vertebrates including humans, two types of OSN, class I and class II, express individual olfactory receptors classified according to amino acid sequence homology [9,10]. Class I OSNs are distributed substantially within zone I, which corresponds to the dorsomedial region of the OE, and their axons converge on glomeruli within the dorsomedial and anterolateral regions (dorsal domain of class I odorant receptors, DI domain) of the olfactory bulb (OB). In contrast, class II OSNs are widely distributed in zones II–IV of the OE, and their axons converge on glomeruli in the dorsolateral (dorsal domain of class II odorant receptors: DII domain) and ventral regions of the OB (ventral domain of class II odorant receptors: V domain) [11]. Since each type of OSN has a different preferred ligand [12], individual types may be activated differently by two olfactory tests that use odors with different chemical structures. Therefore, it is possible to estimate the OSN type (class I or class II) that is activated primarily by the T&T and the IVO odors. Identifying the OSN type impaired in PVOD cases has important implications for therapeutic strategies. Since olfactory stimulation is one of the most effective therapeutic modalities that can promote OE regeneration as a PVOD treatment [13,14], efficient regeneration can be expected if the OE is stimulated with ligands specific for the injured OSN type.

In this study, we examined the characteristics of the IVO and the T&T test response patterns of PVOD cases and compared them with those of individuals with chronic rhinosinusitis (CRS) or post-traumatic olfactory dysfunction (PTOD). We also attempted to identify the OSN type most likely to be impaired in the OB domain of PVOD cases activated by the T&T or the IVO odors. Multivariate analysis of 118 cases of PVOD revealed that a response to the IVO odor, but not to the T&T odors, was a characteristic of PVOD. Furthermore, image analysis of the mouse OB dorsal region revealed that the IVO odor selectively activates glomeruli in the DI domain projected by class I OSNs, whereas the T&T odors primarily activate glomeruli in the DI and DII domains projected by class II OSNs. Combined with the imaging results from mice, data showing responses to the IVO odor rather than the T&T odors may reflect impairment of class I OSNs in the dorsomedial OE, which selectively project their axons to the DI domain of the OB. Thus, OE impairment in PVOD may not be uniform; rather, it may occur heterogeneously in a neuron-type-dependent manner.

## 2. Materials and Methods

### 2.1. Animals

Adult OMP-GCaMP3 transgenic male mice (10 weeks old; Jax #029581, Jackson Laboratories, Bar Harbor, ME, USA) were used for all procedures. Mice were anesthetized with 1–2.5% isoflurane and kept on a heating pad throughout. The bone covering the dorsal OB was thinned and perforated using a dental drill, and a catheter was inserted into the lateral tail vein using a 30-gauge needle connected to a nontoxic medical-grade polyethylene tube (SP10; Natsume Seisakusho Co., Ltd., Tokyo, Japan).

### 2.2. Optical Imaging of GCaMP3 Signals

Glomerular activity in the dorsal region of the OB in mice was evaluated by imaging using olfactometers [15]. First, the T&T test was conducted by stimulating the OE for 10 s with a mixture of five odors (β-phenylethyl alcohol, methyl cyclopentenolone, isovaleric acid, γ-undecalactoneadn, and skatol). These five odors were mixed equally with the highest concentration of stimulant solution used in the T&T test. A custom-made air dilution olfactometer fitted with Teflon tubing was used for odor stimulation; this was controlled by a program written in Labview software (version 9.0.0, National Instruments, MA, USA) based on previous reports [15,16]. Odorant vapor was generated by flowing clean air through a disposable glass microfiber syringe filter (pore size 2.7 μm; Whatman, MA, USA) filled with 30 μL of liquid odorant. The odorant stream (200 mL/min) was mixed with the clean air stream (100 mL/min) in a Teflon tube to produce a total flow rate of 300 mL/min. The odor presentation time was 10 s, and the stimulus interval was at least 60 s (to avoid potential sensory adaptation). The outlet of the tube was placed in front of the nose at a distance of 1 cm. An exhaust tube was placed above the head to suck out any drifting odors. After the experiment using five odor mixtures was completed, the IVO test was conducted by injecting prosultiamine (0.5 mg, Alinamin; Takeda Pharmaceutical Co., Ltd., Osaka, Japan) into the tail vein catheter for a period of 10 s using a syringe pump (KDS Syringe Pump; KD Scientific Inc., Holliston, MA, USA).

Neuronal activity in OSN axon terminals of OMP-GCaMP3 transgenic mice was detected using a Nikon A1R confocal laser scanning microscope system attached to an upright ECLIPSE FN1 microscope (Nikon Corp., Tokyo, Japan) [17]. The fluorescent signal was detected using a 488 nm excitation laser and a 525/50 bandpass emission filter. Sequential images of the OB were acquired at a rate of two frames per s for at least 5 min. Offline analysis was performed using ImageJ software (version 1.53t, NIH, MA, USA) and its plug-in. Photobleaching was corrected for by subtracting the fluorescent changes observed in nonresponsive glomeruli. Since the average size of a murine glomerulus is 100 µm, a circle with a diameter of 100 µm was used as the region of interest. Ca^2+^ responses were calculated as ΔF/F_0_ = (F − F_0_)/F_0_, where F_0_ is the average baseline fluorescence observed before stimulation. Ca^2+^ responses to odor stimulation were performed at least five times during each recording. Excitatory Ca^2+^ responses were defined as significant average increases during the 10 s period after the onset of odor stimulation to the 10 s period before odor stimulation (*p* < 0.05 was considered significant), as previously reported [18].

### 2.3. Immunohistochemical Analyses

Mice were perfused with 4% paraformaldehyde in 0.1% phosphate buffer and postfixed for 24 h in the same fixative. The head tissue including the OE and the OB was decalcified in 10% EDTA solution, pH 7.0, and then embedded in paraffin. Coronal sections were cut (4 μm thick) and mounted on silane-coated slides. For antigen retrieval, deparaffinized sections were autoclaved at 121 °C for 20 min in Target Retrieval Solution (S1700; Dako). Immunohistochemistry was performed using an anti-NQO1 (rabbit polyclonal, 1:300; Cell Signaling Technology) antibody. Immunoreactivity was detected by incubation for 1 h at room temperature with a donkey antirabbit Alexa Fluor 594 (1:100; Invitrogen) antibody.

Serial coronal sections of the OB were prepared; for each section, the DI domain (NQO1-positive region) and the DII domain (NQO1-negative region) were distinguished by the anti-NQO1 antibody [19]. The DI and DII domains were then reconstructed (in three dimensions) from the coronal section to determine the DI and DII domains in the axial section (control sample). The number of responding glomeruli within the DI and DII domains was counted. Glomeruli with an immunostaining intensity exceeding two SDs of the mean background intensity for connective tissue under the lamina propria were considered NQO1-positive.

### 2.4. Study Participants and Analyses Performed for the Clinical Study

This study enrolled 307 patients diagnosed with PVOD, CRS (CRS with polyps and CRS without polyps), or PTOD at the University of Tokyo Hospital between 2014 and 2019. Diagnosis was based on patient history, clinical examination, nasal endoscopy, and CT of the sinuses in accordance with the guidelines of the European Position Paper on Rhinosinusitis and Nasal Polyps [6]. PVOD cases who were examined after 6 months of onset were excluded to avoid the recovery effect. COVID-19-related olfactory dysfunction was not included in the analysis because the mechanism of injury is different from that underlying other types of virus-induced olfactory dysfunction [20]. The olfactory functional tests, the T&T test, and the IVO test were not performed by the same examiner but by a number of examiners according to established testing procedures [21,22].

The T&T test uses the following five odorants [21,23]: (A) β-phenylethyl alcohol, which smells like a rose; (B) methyl cyclopentenolone, which smells like burning sugar; (C) isovaleric acid, which smells like sweat; (D) γ-undecalactone, which smells like a peach; and (E) skatol, which smells like vegetable scraps (Takasago Industry, Tokyo, Japan). The range of concentrations for each odorant spanned eight degrees of intensity (−2–5), except for odorant (B), which spanned seven degrees (−2–4). These odorants were presented to the entrance of the participant’s nostrils to orthonasally stimulate the OSNs. The recognition threshold was defined as the lowest concentration at which the odor could be identified. Subsequently, the recognition threshold for five odorants was averaged, and olfactory function was evaluated based on these averaged values. If all odors were recognized at the minimum concentration, the averaged value would be −2, while if none of the odors were recognized, the averaged value would be 5.8. No response was defined as an averaged vale of ≥5.6 for the five odors, while a value of <5.6 was defined as a response.

The IVO test, which is a retronasal olfactory test that can be performed without age restriction as long as the patient can communicate, involves the stimulation of OSNs by the intravenously-administered odorant prosultiamine (Alinamin, Takeda Pharmaceutical Company Limited, Osaka, Japan) [8]. Briefly, prosultiamine (10 mg; 2 mL) was injected into an antecubital vein at a constant rate over 20 s. Onset was defined as the first report of a definite smell of garlic/onion. The duration of smell sensation from initiation to termination was also measured. Based on previous data in the healthy groups, less than 10 s was considered to be a normal onset time, and more than 60 s was considered to be normal duration [7,22,24]. In this study, a response was defined as the perception of a garlic odor after administration, and no response was defined as no perception of garlic odor within 3 min. The following classification was made according to the presence or absence of a response to the two tests: T&T(+) IVO(+) if the patient responded to both odors, T&T(−) IVO(−) if the patient did not respond to either odor, T&T(+) IVO(−) if the patient responded only to the T&T odors, and T&T(−) IVO(+) if the patient responded only to the IVO odor.

Univariate and multivariate logistic regression analyses were performed to identify factors (as dependent variables) predictive of PVOD, T&T(+) IVO(+), T&T(−) IVO(+), T&T(+) IVO(−), and T&T(−) IVO(−). In the logistic regression analysis, males were coded as 1 and females as 0. Associations between predictive factors and dependent variables were expressed as odds ratios (OR) and respective 95% confidence intervals (95% CI). Variables with a *p*-value < 0.05 in univariate analysis were included in multivariable logistic regression analysis.

### 2.5. Statistical Analyses

Statistical analyses were performed using the Mann–Whitney *U*-test and the Chi-square test.

## 3. Results

The response patterns of PVOD, CRS (CRS with polyp and CRS without polyps), and PTOD cases, diagnosed at the University of Tokyo Hospital over a 6 year period from 2014 to 2019, were recorded after the T&T and IVO tests. Of the 171 PVOD cases, 37 for whom more than 6 months had elapsed since onset were excluded. Of the remaining 134 cases, 17 who underwent only one olfactory functional test (T&T or IVO) were excluded. The remaining 118 cases (22 cases of influenza and 96 cases of infection by an unknown viral species) were analyzed. Of the 387 cases of CRS with olfactory dysfunction, 173 were evaluated using both olfactory tests. Of these, 12 cases that underwent only one olfactory test were excluded; the remaining 161 cases were analyzed. Of the remaining cases, 117 were categorized as CRS with polyps, and 44 cases were categorized as CRS without polyps (in accordance with the guidelines of the European Position Paper on Rhinosinusitis and Nasal Polyps) [6]. Of the 60 PTOD cases, 22 cases for whom more than 6 months had elapsed since the onset were excluded. Of the remaining 38 cases, 10 who underwent only one olfactory test were excluded, and the remaining 28 cases were analyzed. Disease type (PVOD, CRS with polyps, CRS without polyps, and PTOD), period to examination, age, sex, and the results of the IVO and T&T tests are shown in Table 1.

**Figure 1:**
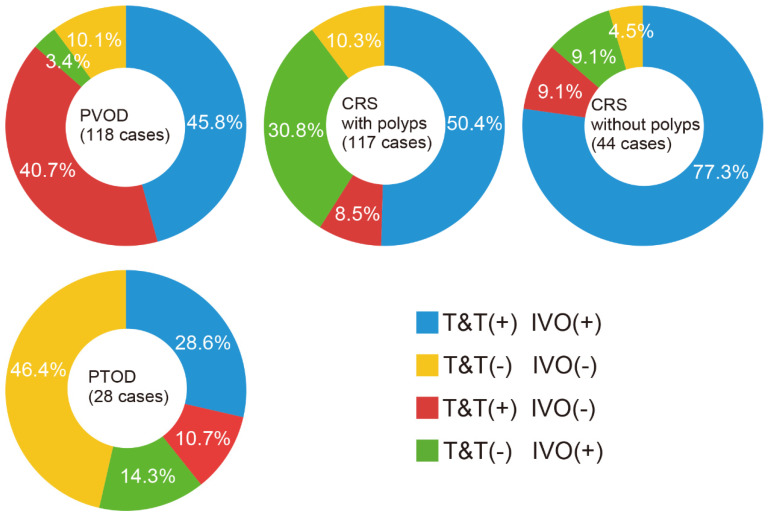
Summary of the test results in cases with PVOD (post-viral olfactory dysfunction), CRS with polyps (chronic rhinosinusitis with polyps), CRS without polyps (chronic rhinosinusitis without polyps), and PTOD (post-traumatic olfactory dysfunction). Based on the results of the two olfactory functional tests (T&T and IVO tests), responses were classified into four types: T&T(+) IVO(+), reaction to both T&T and IVO odors; T&T(−) IVO(−), no reaction to either T&T or IVO odors; T&T(+) IVO(−), reaction only to T&T odors; T&T(−) IVO(+), reaction only to the IVO odor.

The results of the two tests were classified as responsive or nonresponsive; the test results for PVOD, CRS with polyps, CRS without polyps, and PTOD are shown in Figure 1. Of the 118 cases with PVOD, 54 (45.8%) were responsive to both odors, 48 (40.7%) to the T&T odors only, four (3.4%) to the IVO odor only, and 12 (10.1%) to neither. Of the 117 cases with CRS with polyps, 59 cases (50.4%) responded to both odors, 10 (8.5%) to the T&T odors only, 36 (30.8%) to the IVO odor only, and 12 (10.3%) to neither. Of the 44 cases with CRS without polyps, 34 (77.3%) responded to both odors, four (9.1%) to the T&T odors only, four (9.1%) to the IVO odor only, and two (4.5%) to neither. Of the 28 cases with PTOD, eight (28.6%) responded to both odors, three (10.7%) to the T&T odors only, four (14.3%) to the IVO odor only, and 13 (46.4%) to neither. We examined the most effective combinations that predict PVOD by first conducting univariate logistic regression analysis to select the appropriate parameters and then by entering the relevant values into the multivariable logistic regression analysis (Table 2).

Univariable analysis identified sex, T&T(+) IVO(+), T&T(−) IVO(+), and T&T(+) IVO(−) as being predictive of PVOD (Table 2). Multivariate logistic regression analysis including sex, T&T(+) IVO(+), T&T(−) IVO(+), and T&T(+) IVO(−), identified female sex and the combination T&T(+) IVO(−) as significant positive predictors of PVOD (OR, 6.9; sensitivity, 41%; specificity, 91%), while the combination T&T(−) IVO(+) was a significant negative predictor of PVOD (Table 2). The combination T&T(+) IVO(+), although frequent among PVOD, had low specificity and was not a significant predictor of PVOD (*p* = 0.04, OR, 1.4; sensitivity, 54%; specificity, 53%).

Next, we examined the type of olfactory disorder predicted by the combined response patterns. Univariate analysis revealed that the combination T&T(+) IVO(+) was predictive of CRS w/o polyps and PTOD (Table 3). Multivariate analysis identified the combination T&T(+) IVO(+) as predictive of CRS without polyps (OR, 4.0; sensitivity, 77%; specificity, 54%, Table 3).

The combination T&T(−) IVO(+) was a significant predictor of PVOD and CRS with polyps (Table 4). Multivariate analysis of these two diseases showed that T&T(−) IVO(+) was a significant positive predictor of CRS with polyps (OR, 6.6; sensitivity, 31%; specificity, 94%, Table 4), while T&T(−) IVO(+) was a significant negative predictor of PVOD (Table 4).

Sex, PVOD, CRS with polyps, and CRS without polyps were significant factors predictive of a T&T(+) IVO(−) response (Table 5). Multivariate analysis of these three diseases showed that only PVOD was predicted by a T&T(+) IVO(−) response (OR, 6.9; sensitivity, 41%; specificity, 91%, Table 5).

The combination T&T(−) IVO(−) was the only significant predictor of PTOD (OR, 8.4; sensitivity, 46%; specificity, 91%, Table 6). Taken together, the results indicate that PVOD is characterized by a response to T&T(+) IVO(−) and female sex; conversely, T&T(+) IVO(−) strongly predicts PVOD.

### Imaging of the Mouse OB

**Figure 2:**
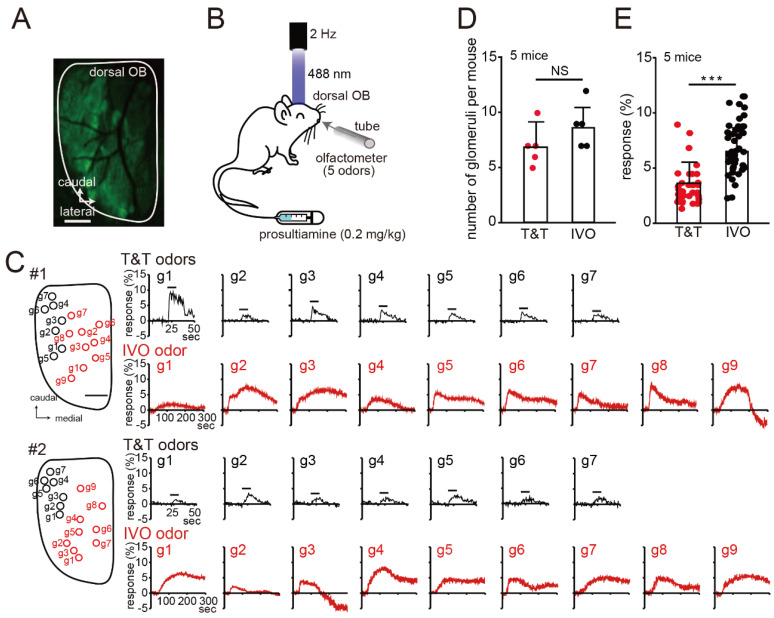
Neural responses of the murine dorsal OB to stimulation with the T&T and the IVO odors. (**A**) Confocal image of the GCaMP3 signals before odor stimulation. Scale, 500 μm. (**B**) Schematic of the experimental design. Glomerular activity in the dorsal region of the murine OB was evaluated by imaging using olfactometers. First, the OE was stimulated with a mixture of the five odors used in the T&T test. Next, prosulthiamine was administered via the tail vein (0.2 mg/kg). (**C**) Left, images of the OB dorsal surface on the right side. g1–g7 are the individual glomeruli (black, glomerulus activated by the T&T odors; red, glomerulus activated by the IVO odor). Scale bar, 500 μm. Right, odorant-evoked Ca^2+^ responses in the target glomerulus. Black bars show the timing of odorant delivery (10 s). (**D**) Summary of the number of glomeruli activated by the T&T and the IVO odors. There were no significant differences between the number of glomeruli activated by the IVO odor and that activated by the T&T odors. Mann–Whitney test, NS, not significant, n = 5 mice. (**E**) Summary of the neural responses to stimulation with the T&T and IVO odors (T&T odors, 35 glomeruli; IVO odor, 44 glomeruli). The neural response to stimulation with the IVO odor was significantly greater than that to stimulation with the T&T odors. Mann–Whitney test, *** *p* < 0.001, n = 5 mice.

Odor responses to the T&T and IVO odors in the dorsal region of the mouse OB were recorded to infer the pathogenesis of PVOD with T&T(+) IVO(−) (Figure 2A). An olfactometer was used to stimulate OE with a mixture of five odors (T&T odors, Figure 2B) [16]. The T&T odors preferentially activated glomeruli in the lateral posterior region of the OB (#1 and #2 mouse, Figure 2C). After the experiment using the T&T odors, prosulthiamine (IVO odor) was injected via the tail vein, and neural responses of the alveoli via the posterior nostril were recorded. Neural responses to stimulation with the IVO odor resulted in extensive activation of the dorsal regions (#1 and #2 mouse, Figure 2C). There was no significant difference in the number of glomeruli responding to the T&T and IVO odors (N = 5 mice; Mann–Whitney test, *p* = 0.19; Figure 2D). In addition, the neural response to stimulation with the IVO odor was significantly stronger than that in response to stimulation with the T&T odors (T&T odors, 35 glomeruli; IVO odor, 44 glomeruli; n = 5 mice, Mann–Whitney test, *** *p* < 0.001; Figure 2E). These results suggest that the lack of response to the IVO odor but a response to the T&T odors is not caused by the activation of very few glomeruli or a weak stimulus.

**Figure 3:**
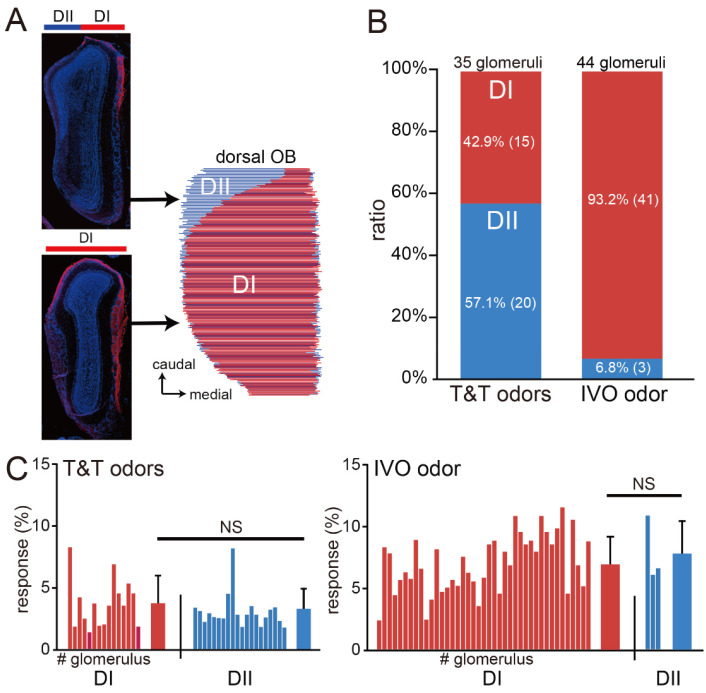
The T&T odors activate the DI and DII domains, while the IVO odor preferentially activates the DI domain. (**A**) Distribution of the DI and DII domains in the dorsal OB. The 3D reconstruction from coronal tissue sections identified each domain in the dorsal OB. Red, anti-NQO1 antibody; blue, DAPI. (**B**) Summary of the glomerular regions activated by the T&T and IVO odors. The T&T odors activated the DI and DII domains almost equally, but most glomeruli activated by the IVO odor belonged to the DI domain. (**C**) Summary of the intensity of the response of the D1 and DII domains to stimulation with the T&T (**left**) and the IVO odors (**right**). The horizontal axis lists the glomeruli that responded, and the bar graph on the right represents the mean values; there were no significant differences between the DI and DII domains with respect to the response intensity to the T&T odors (**left**). Similarly, there were no significant differences between the DI and DII domains with respect to response intensity to the IVO odor (**right**). Mann–Whitney test, NS, not significant.

To identify the OB domains to which the responding glomeruli belonged, we prepared coronal sections of mice and immunostained them to distinguish the DI (NQO1-positive) and DII (NQO1-negative) domains. The OB coronal sections were then reconstructed in 3D to examine how the DI and DII domains were distributed on the dorsal surface exposed during the imaging experiments (Figure 3A). We found that the T&T odors stimulated glomeruli in the DI and DII domains almost equally, while the IVO odor activated glomeruli mainly in the DI domain (T&T odors, 35 glomeruli; IVO odor, 44 glomeruli, n = 5 mice; Chi-square test, *p* < 0.001; Figure 3B). Furthermore, there was no significant difference in the response intensity between the DI and DII glomeruli activated by the T&T odors (DI domain, 15 glomeruli; DII, 20 glomeruli; Mann–Whitney test, *p* = 0.32; Figure 3C). Similarly, there was no significant difference between the DI and DII glomeruli with respect to the intensity of the response activated by the IVO odor (DI domain, 40 glomeruli; DII, 4 glomeruli; Mann–Whitney test, *p* = 0.31; Figure 3C).

These results suggest that differences in the activated domains are caused by differences in odor molecules rather than differences in stimulus intensity. Thus, the combination that is unresponsive to the IVO odor but responsive to the T&T odors implies reduced neural activity of class I OSNs with axonal projections selectively in the DI domain, suggesting that in the PVOD cases, the reduction in neural activity occurs heterogeneously within the OE in a neuron-type-dependent manner.

## 4. Discussion

In this study, we focused on PVOD cases and examined their characteristic responses to the IVO and T&T tests. Multivariate analysis revealed that PVOD is characterized by a response to the IVO odor but not to T&T odors. Image analysis of the mouse OB revealed that the T&T odors activated a wide range of glomeruli spanning the DI domain projected by class I OSNs and the DII domain projected by class II OSNs, whereas the IVO odor mainly activated glomeruli in the lateroposterior region (corresponding to the DI domain).

A variety of methods and large systematic panels of odorants have been used to map odorant-evoked glomerular activity in the mammalian OB [25,26,27,28]. These studies clearly show that individual odorants activate a specific combination of glomeruli and reveal the molecular receptive range (MRR) of individual glomeruli as well as spatially mapping the MRR on the glomerular sheet in the dorsal surface of the OB [11,26,28,29]. For example, glomeruli activated by a linear fatty acid containing isovaleric acid (a T&T odor) as well as a linear amine containing prosultiamine (an IVO odor) are localized in the DI domain. In mammals, most odorant receptors whose ligands are aliphatic compounds (e.g., relatively hydrophilic acids and amines) belong to class I OSNs, which project to the D1 domain [30]. In contrast, glomeruli activated by alcohols, which include β-phenylethyl alcohol (a T&T odor) and linear ketones that include γ-undecalactone (a T&T odor), are localized in the DII domain [11,26,28,29]. In short, T&T odors activate a wide range of glomeruli in the OB including the DI and DII domains, whereas the IVO odor is expected to activate glomeruli selectively within the DI domain. Consistent with these reports, our experiments showed that 93.2% of the glomeruli activated by the IVO odor belonged to the DI domain, whereas 42.9% and 57.1% of those activated by the T&T odors belonged to the DI and DII domains, respectively. Close examination of surgically removed human nasal specimens revealed an OE with clusters of mature OSNs in the posterior superior part of the nasal cavity, similar to that observed in mice [31]. Furthermore, the human OE expresses class I and class II OSNs [32], as in mice. Although human OBs contain more glomeruli than those of mice, their anatomical organization and the molecular mechanisms underlying olfactory information processing are considered comparable with those described in rodents, suggesting the general preservation of functional properties [33]. Thus, the lack of response to the IVO odor suggests reduced neural activity in the glomeruli of the DI domain. Since class I OSNs in the dorsomedial OE selectively project their axons to the glomeruli of the DI domain, class I OSNs in the dorsomedial OE could be strongly impaired in PVOD, and there would be a difference in susceptibility to injury within the OE.

It is interesting to consider that intracellular enzyme activity may result in differences in OSN susceptibility to injury. Class I OSNs are characterized by the presence of NQO1, a cytosolic flavoenzyme that catalyzes the 2-electron reduction of quinones and aromatic nitro compounds. Essentially, NQO1 protects cells against quinone toxicity; however, NQO1 bioactivation within metabolic pathways involving multiple enzymes is not simple [34]. In particular, conjugation of NQO1 to manganese superoxide dismutase (MnSOD), a primary antioxidant enzyme located in mitochondria, mediates the generation of reactive oxygen species (ROS) via the reaction of unstable hydroquinones with oxygen, leading to oxidative stress [34,35,36]. Viral infection induces various inflammatory cytokines in the OE [37,38], and secretion of these cytokines induces the expression of MnSOD [39]. Therefore, viral infection may trigger an increase in ROS production in the OE, causing damage to class I OSNs.

Identification of the OSN type most likely to be impaired in PVOD is of great importance in the context of therapeutic strategies. Olfactory input is an important factor involved in regeneration of the OE in mouse and humans. In mice, after methimazole-induced damage to the OE when olfactory input is blocked by silicon tube insertion, new OSNs fail to mature and instead undergo apoptosis [14]. A study of 70 human patients with PVOD reported a significant improvement in olfactory function when sniffing four odors at high concentrations for 18 weeks compared with a group sniffing four odors at low concentrations [13]. Preferential use of linear fatty acids and linear amines, which are ligands for class I OSNs, as a therapeutic panel could promote efficient regeneration of class I OSNs, leading to early improvement of olfactory perception in PVOD.

In Japan, two olfactory function tests are covered by health insurance and are performed widely at many clinics. The T&T test uses five different odors to activate OSNs and estimates the degree of olfactory dysfunction by calculating the average cognitive threshold for the five different odors. In contrast, the IVO test activates OSNs using a single odor. The degree of OE damage is estimated by measuring the time from the administration of prosultiamine to perception of the odor (latency), and the time that the odor is perceived (duration).

To date, no study has combined two conventional methods, each with different characteristics (i.e., the detection and recognition thresholds for the T&T test, and the latency and duration for the IVO test) to examine the etiology of olfactory dysfunction. This study is the first to attempt to distinguish the etiology of olfactory dysfunction using a combination of the presence or absence of responses to these two tests.

The combination T&T(+) IVO(−) predicted PVOD with high specificity. The lack of response to IVO odor, which selectively activates class I OSNs, suggests the possibility of heterogeneous damage to the OE. Even in cases without a clear history of the common cold, a T&T(+) IVO(−) response may be a reason to suspect PVOD. The combination T&T(−) IVO(+) predicted CRS with polyps with high specificity. In CRS with polyps, nasal polyps tend to form in the middle meatus, reducing airflow from the superior meatus toward the olfactory cleft. However, airflow in the direction of the olfactory cleft is relatively preserved upon stimulation via the retronasal pathway [8]. Reflecting this pathology, CRS with polyps may be characterized by a combination that shows no response to the T&T odors, but a response to the IVO odor. The combination T&T(+) IVO(+) predicted CRS without polyps with high specificity. However, CRS involves more complex heterogeneous inflammatory processes including restricted mucociliary clearance [40], a direct antipathogenic effect [41], and abnormalities in the sinonasal epithelial cell barrier [42]. Furthermore, ECP, a degranulated protein from eosinophils, impairs OSNs directly. Therefore, in CRS with olfactory neuropathy, it may be difficult to predict disease status based solely on the presence or absence of a response to the two tests. Since the degree of latency on the IVO test correlates with the degree of OE damage [8], measuring latency and the average cognitive threshold in the T&T test may be useful for estimating the degree of OE damage. The combination T&T(−) IVO(−) predicted PTOD with high specificity. In PTOD, olfactory deficits are often caused by mechanisms such as direct damage to OSN axons at the cribriform plate and hemorrhage within the olfactory cortex [43]. The degree of olfactory dysfunction depends on factors such as the severity of the head injury, the location of the trauma, and the duration of post-traumatic amnesia [44,45], but in general, it takes considerable time for the sense of smell to recover, and in some cases, the sense of smell does not recover at all [46,47]. Reflecting these pathologies, PTOD cases may be characterized by a lack of response to both T&T and IVO odors, which may reflect more severe OE injury and poor prognosis.

In this study, the five T&T odors were grouped together to define the presence or absence of a response; however, in clinical practice, responses to each of the five odors are recorded separately. Therefore, a combined analysis of the responses to the individual odors comprising the T&T odors as well as the responses to the IVO odor may help to more accurately estimate the characteristics of OE injury in future studies.

The current study had several limitations. First, it was a retrospective cohort design and was subject to the inherent biases that come with these types of studies. Second, although there are many causes of olfactory dysfunction, this study only analyzed the top three causes. Third, our study was performed on a limited number of patients with PVOD, and all patients presented at a single institution. Therefore, the generalizability of our findings to other settings is unclear. Fourth, due to technical reasons, optical mapping of glomerular activity was limited to the dorsal surface of the OB. However, even when only the dorsal region was considered, there were differences in the domain preference of each odor panel, strongly suggesting that OE may be injured heterogeneously in PVOD. Overall, this is the first study to demonstrate the possibility of class I OSN-dominant injury in PVOD based on characteristic response patterns to olfactory tests.

## 5. Conclusions

The characteristics of the response patterns of PVOD cases were examined using the IVO and T&T tests, which use different odors. A nonresponse to the IVO odor and a response to the T&T odors was characteristic of PVOD. Image analysis of mouse OBs revealed that the IVO odor selectively activated the DI domain, into which the class I OSNs projected their axons, while the T&T odors broadly activated the DII domain, into which the class II OSNs projected their axons, in addition to the D1 domain. Thus, class I OSNs could be injured preferentially in PVOD.

## Figures and Tables

**Table 1:** Patient demographics (N = 307).

Disease Type	N	Period to Exam. (Months)	Age (y)	Sex (M/F)	IVO Test			T&T Test	
					Response (Yes/No)	Latency (s)	Duration (s)	Response (Yes/No)	Recognition Score
PVOD	118	3.0 ± 1.5	54.1 ± 12.5	M: 26 (22%) F: 92 (78%)	58 (49%) /60 (51%)	22.1 ± 10.5	71.3 ± 46.9	102 (86%) /16 (14%)	3.6 ± 1.7
CRS with polyps	117	n.d.	54.4 ± 10.2	M: 79 (68%) F: 38 (32%)	95 (81%) /22 (19%)	18.9 ± 7.8	66.9 ± 35.3	69 (59%) /48 (41%)	4.4 ± 1.5
CRS without polyps	44	n.d.	52.1 ± 9.2	M: 30 (68%) F: 14 (32%)	39 (89%) /5 (11%)	15.5 ± 7.9	80.6 ± 51.8	37 (84%) /7 (16%)	3.3 ± 1.7
PTOD	28	3.0 ± 1.6	46.3 ± 12.6	M: 14 (50%) F: 14 (50%)	12 (43%) /16 (57%)	17.3 ± 7.3	55.9 ± 30.4	11 (39%) /17 (61%)	5.2 ± 1.1

PVOD, post-viral olfactory dysfunction; PTOD, post-traumatic olfactory dysfunction; CRS, chronic rhinosinusitis; w/o, without; N, number of cases; Exam., examination; n.d., not determined; M, male; F, female.

**Table 2:** Univariate and multivariate analysis of the factors predictive of PVOD (N = 118).

	Variable	N	Univariable Coefficient (95%CI)	*p*-Value	Multivariable, OR (95%CI)	*p*-Value
PVOD	Age		0.01 (−0.01–0.03)	0.32		
	Sex	M: 26 F: 92	−2.0 (−2.52–(−1.55))	<0.001	0.13 (0.07–0.24)	<0.001
	T&T(+) IVO(+)	54	−0.42 (−0.84–(−0.01))	0.04	1.35 (0.59–3.1)	0.48
	T&T(−) IVO(+)	4	−2.27 (−3.3–(−1.25))	<0.001	0.22 (0.06–0.81)	0.02
	T&T(+) IVO(−)	48	2.15 (1.63–2.67)	<0.001	8.42 (3.14–22.56)	<0.001
	T&T(−) IVO(−)	12	−0.07 (−0.75–0.6)	0.83		

PVOD, post-viral olfactory dysfunction; N, number of cases; M, male; F, female; CI, confidence interval; OR, odds ratio.

**Table 3:** Univariate/multivariate analysis of the factors predictive of T&T(+) IVO(+) (N = 155).

	Variable	N	Mean	Univariate Coefficient (95% CI)	*p*-Value	Multivariate, OR (95% CI)	*p*-Value
T&T(+) IVO(+)	Age	155	52.8 ± 10.8	−0.01 (−0.02–0.02)	0.77		
	Sex	M: 77 F: 78		0.09 (−0.36–0.54)	0.69		
	PVOD	54		−0.31 (−0.77–0.15)	0.19		
	CRS with polyps	59		−0.01 (−0.46–0.46)	0.99		
	CRS without polyps	34		1.38 (0.64–2.13)	<0.001	3.67 (1.73–7.77)	<0.001
	PTOD	8		−1.02 (−1.88–(−0.17))	0.02	0.43 (0.18–1.02)	0.56

PVOD, post-viral olfactory dysfunction; CRS, chronic rhinosinusitis; PTOD, post-traumatic olfactory dysfunction; N, number of cases; M, male; F, female; CI, confidence interval; OR, odds ratio.

**Table 4:** Univariate/multivariate analysis of the factors predictive of T&T(−) IVO(+) (N = 48).

	Variable	N	Mean	Univariate Coefficient, (95% CI)	*p*-Value	Multivariate, OR (95% CI)	*p*-Value
T&T(−) IVO(+)	Age		53.8 ± 10.1	0.01 (−0.02–0.03)	0.61		
	Sex	M: 29 F: 19		0.57 (−0.06–1.20)	0.08		
	PVOD	4		−2.16 (−3.21–(−1.10))	<0.001	0.28 (0.08–0.97)	0.04
	CRS with polyps	36		1.89 (1.18–2.59)	<0.001	3.56 (1.55–8.18)	0.003
	CRS without polyps	4		−0.69 (−1.78–(−0.38))	0.2		
	PTOD	4		−0.12 (−1.22–0.99)	0.84		

PVOD, post-viral olfactory dysfunction; CRS, chronic rhinosinusitis; PTOD, post-traumatic olfactory dysfunction; N, number of cases; M, male; F, female; CI, confidence interval; OR, odds ratio.

**Table 5:** Univariate and multivariate analysis of the factors predictive of T&T(+) IVO(−) (N = 65).

	Variable	N	Mean	Univariate Coefficient (95% CI)	*p*-Value	Multivariate, OR (95%CI)	*p*-Value
T&T(+) IVO(−)	Age		54.1 ± 12.5	0.02 (−0.02–0.04)	0.35		
	Sex	M: 25 F: 40		0.89 (0.004–1.78)	0.04	1.52 (0.76–3.03)	0.24
	PVOD	48		1.94 (1.32–2.56)	<0.001	6.52 (1.82–23.42)	0.004
	CRS with polyps	10		−1.47 (−2.19–(−0.75))	<0.001	0.73 (0.18–2.85)	0.65
	CRS without polyps	4		−1.11 (−2.17–(−0.04))	0.04	0.77 (0.16–3.78)	0.75
	PTOD	3		−0.87 (−2.10–0.36)	0.17		

PVOD, post-viral olfactory dysfunction; PTOD, post-traumatic olfactory dysfunction; CRS, chronic rhinosinusitis; N, number of cases; M, male; F, female.

**Table 6:** Univariate analysis of the factors predictive of T&T(−) IVO(−) (N = 39).

	Variable	N	Mean	Univariate Coefficient (95% CI)	*p*-Value
T&T(−) IVO(−)	Age		53.2 ± 13.5	0.01 (−0.03–0.03)	0.89
	Sex	M: 18 F: 21		−0.11 (−0.78–0.56)	0.75
	PVOD	12		−0.39 (−1.11–0.34)	0.29
	CRS with polyps	12		−0.37 (−1.09–0.35)	0.31
	CRS without polyps	2		−1.23 (−2.7–(−0.23))	0.09
	PTOD	13		2.13 (1.29–2.98)	<0.001

PVOD, post-viral olfactory dysfunction; CRS, chronic rhinosinusitis; PTOD, post-traumatic olfactory dysfunction; N, number of cases; M, male; F, female; CI, confidence interval; OR, odds ratio.

## Data Availability

The data presented in this study are available on request from the corresponding author. To protect privacy, the data are not publicly available.
